# Effect of human follicle-stimulating hormone on immunomodulatory function of decidual mesenchymal stem cells by reducing interleukin-6 levels

**DOI:** 10.1186/s13048-022-00993-3

**Published:** 2022-05-13

**Authors:** Yi-bo He, Li Zhang, Lin-li Zhou, Yi-min Chen, Jia-hong Lu, Jie Chen, Yong-lin Liu

**Affiliations:** 1 Department of Clinical Lab, The First Affiliated Hospital of Zhejiang Chinese Medical University, 54 Youdian Road, Hangzhou, 310006 China; 2 Obstetrics and Gynecology, The First Affiliated Hospital of Zhejiang Chinese Medical University, 54 Youdian Road, Hangzhou, 310006 China; 3 Department of Clinical Lab, The Third District of the Chinese People’s Liberation Army Air Force Hangzhou Special Service Rehabilitation Center, 76 Yuhuangshan Road, Hangzhou, 310012 China; 4 Obstetrics and Gynecology, The First People’s Hospital of Xiaoshan District, 199, Xinnan Road, HangzhouHangzhou, 311200 China; 5 Department of Endocrinology, The First Affiliated Hospital of Zhejiang Chinese Medical University, 54 Youdian Road, 310006 Hangzhou, China; 6 Reproductive Centre, Sanya Women and Children’s Hospital Managed By Shanghai Children’s Medical Center, 339 Yingbin Road, Sanya, 572000 China

**Keywords:** Mesenchymal stem cells, Interleukin-6, Follicle stimulating hormone, Myeloid differentiation factor 88

## Abstract

**Objective:**

Women with an elevated basal FSH indicate diminished ovarian reserve and reduced oocyte and embryo numbers. DMSCs are likely to be involved in immune tolerance of pregnancy maintenance. We investigate the effect of follicle-stimulating hormones on the immunomodulatory functions of DMSCs.

**Methods:**

DMSCs were primary cultured from decidual tissue. Pretreated DMSCs with mitomycin C, combined with CD4^+^ T lymphocytes, DMSCs + CD4^+^T co-culture system was established. Different physiological dose FSH (3 ng/ml,10 ng/ml,30 ng/ml,100 ng/ml) were used to co-culture system. Cytokines (IFN-γ, IL-2, IL-4, IL-6, IL-10, TNF-α) and other proteins (FSHR, MyD88) were measured.

**Results:**

Compared with the control group (FSH (0 ng/mL) + CD4^+^T + DMSCs), the FSH concentration was 10, 30, and 100 ng/ml, IL-6 levels were significantly reduced (*P* < 0.05). IL-6, MyD88 protein expression was remarkably decreased (*P* < 0.05).

**Conclusion:**

FSH/FSHR could negatively regulate the immunosuppressive function of DMSCs by reducing secretion of IL-6 levels through MyD88 pathways, but upstream and downstream signalling pathways require further validation.

**Supplementary Information:**

The online version contains supplementary material available at 10.1186/s13048-022-00993-3.

## Introduction

Mesenchymal stem cells (MSCs) are adult stem cells with significant self-renewal ability and multilineage differentiation capacity [1]. MSCs can be isolated from various tissues, including bone marrow, adipose tissue, umbilical cord, and others [2]. Decidua is also an essential source of MSCs [3]. Some studies indicate that decidual mesenchymal stem cells (DMSCs) have a wide range of immune regulation functions better than bone marrow-derived MSCs [4]. Researches show that Human Decidua plays an essential role in Immune Tolerance to maintain normal pregnancy [5–7]. Dendritic cells, natural killer cells, T cells, macrophages, and other immune cells in the decidual tissue constitute the immune microenvironment to maintain pregnancy stability. MSCs have an immune regulation function and can maintain immune tolerance [6], A variety of MSCs could be isolated from term placental tissue, including DMSCs [7]. As an essential component of decidual cells, DMSCs were likely to be involved in immune tolerance of pregnancy maintenance [8].

There are many cytokines in the microenvironment, including Th1 and Th2 cytokines. Th1 cytokines are detrimental to implantation, trophoblast cell growth, and embryonic development, while Th2 cytokines inhibit Th1 cytokines from preventing secondary damage to trophoblast cells and fetus[9]. Follicle-stimulating hormone (FSH), a glycoprotein hormone family member, promotes and maintains normal gonadal development and reproductive function. Besides, FSH can participate in immune regulation by mediating the secretion of immune factors through follicle-stimulating hormone receptors (FSHR) distributed in gonads. BMSCs have been found to express FSHR [10]. Several studies suggest a negative effect of raised FSH on the quality of embryos and in vitro fertilization (IVF) treatment outcome [11–13]. Women with an elevated basal FSH indicate diminished ovarian reserve and reduced numbers of oocytes and embryos [14, 15]. Therefore, we speculate that the reason might be related to FSH regulating the immune function of DMSCs.

At present, there is no relevant report on the regulatory effect of FSH on immune function of DMSCs. It may help to understand the role of DMSCs in embryo implantation, immune tolerance, and pregnancy maintenance and provide a research basis for the pathogenesis research and treatment of abnormal pregnancy such as threatened abortion and missed abortion.

## Materials and methods

### Primary culture of DMSCs

Samples of human decidual tissue (ten samples) were collected from healthy women aged 20–30 years undergoing elective vaginal surgical termination of early pregnancy (6–8 gestational weeks). In the laboratory, every sample should be washed three times in DMEM/F12 (Genom Biotechnology, China) containing antibiotics (100 IU/ml penicillin and 100 mg/mL streptomycin). Samples were thoroughly separated from the trophoblast and minced carefully into about one mm^3^ piece by Surgical scissors. After washing with PBS (pH 7.4), decidual tissue was incubated in 0.25% collagenase type I and IV 1:1 (GIBCO, USA) at 37℃ for 80 min. The cell suspension was filtered through Stainless steel filter (70 mm). Then, 4 ml 0.84% NH_4_CL for lysing the red blood cells in a four ℃ refrigerator for 15 min. After washing PBS (pH 7.4) twice, draw 6 ml into a 25 cm^2^ cell-culture flask, add DMEM / f12 with 10% fetal bovine serum and 2 ml/l glutamate (Sigma, Germany). Cells were grown in 37C, 5% CO_2_, and 95% humidity cell incubator. Fourth to sixth passage cells were used in all experiments; cells were observed under a microscope and photographed.

### Phenotypic analysis

After the primary cells were collected, the concentration was adjusted to 1 × 10^5^/ml, and PBS was added to prepare the suspension. The cultured DMSCs were measured by Flow cytometry with antibodies against specific markers for mesenchymal cells. The antibodies, Anti-CD45-FITC、Anti-CD34-PE, Anti-HLA-DR-PE, Anti-CD29-PE, Anti-CD79-PE, Anti-CD90-FITC, Anti-CD146-PE, Anti-CD19-ECD, Anti-CD73-PE, Anti-CD3-FITC(Beckman coulter, USA)were added in DMSCs suspensions. The specific fluorescent labelling of 10,000 events was analyzed in a Beckman coulter FACSCalibur flow cytometer using Kaluza 2.1 software (Beckman coulter, USA). Each experiment was repeated three times, and each experiment included three replicates. The average values of triplicate measurements were used as each activity value.

### Differentiation ability of DMSCs as osteogenic and adipogenic cells

Configuration of osteogenic cells induction medium: high glucose DMEM/F12 (Genom Biotechnology, China), 2 mM glutamine (Genom Biotechnology, China), 100 nM dexamethasone (Sigma, Germany), 0.2 mm ascorbic acid (Sigma, Germany), 10 mM to-sodium glycerophosphate (Sigma, Germany). Cells were cultured in an osteogenic cells induction medium for 20 days. Osteogenic differentiation of DMSCs was assessed using alizarin red staining (Merrybaugh, China).

Configuration of Adipogenic Cells induction medium: solution A: high glucose DMEM/F12 (Genom Biotechnology, China), 2 mM glutamine (Genom Biotechnology, China), one mol/L dexamethasone (Sigma, Germany), 10 g/ml bovine insulin (GIBCO, USA), 0.5 mM IBMX (Sigma, Germany), 200 M Indomethacin (Sigma, Germany). Solution B: high glucose DMEM/F12 (Genom Biotechnology, China), 1 M dexamethasone (Sigma, Germany), 10 g/ml bovine insulin (GIBCO, USA). After three days in culture in solution A, the induction medium was replaced with Solution B, then culture for 20 days. Adipogenic differentiation was assessed using oil red O stain (Sigma, Germany).

### Expression of FSHR mRNA and protein in DMSCs

Total RNA was isolated from DMSCs by using RNeasy mini kit (Servicebio, China). Real-time PCR was performed using the RT system according to the manufacturer's instructions. The PCR program was as follows: hold at 98 °C for 5 min followed by at 98 °C for 15 s, at 55 °C for 15 s and at 72 °C for 30 s for 35 cycles. The primer sequences of human FSHR were as follows: the upper primer 5 tcaggctaggggtcagagat-3, the lower primer 5'-gctcaccttcatgtagctgc-3', the expected fragments were 631 bp.

DMSCs were fixed in 4% paraformaldehyde in PBS at 4 °C for 15 min, and cells were embedded in paraffin and sectioned. Fixed samples were incubated with 0.1% Triton X-100 in PBS for 5 min and BlockingOne (Servicebio, China) for 30 min, followed by anti-FSHR (Servicebio, China) at a dilution of 1:500 in 5% BlockingOne at 4 °C overnight. Cells were washed 3 times with 0.1% Tween 20 in PBS and incubated with Alexa Fluor 488-coupled anti-rabbit secondary antibody (Servicebio, China) at a dilution of 1: 500. Cells were washed with DAPI (Servicebio, China). Images were collected and processed by fluorescence microscopy (Nikon, Japan). The average values of triplicate measurements were used as each activity value.

### Effect of FSH on proliferation and apoptosis of DMSCs

DMSCs were seeded at a density of 5000 cells per well in 96-well plates after adding FSH at 0 ng/ml, 3 ng/ml, 10 ng/ml, 30 ng/ml, 100 ng/ml. The plate was returned to a 5% CO_2_ incubator at 37 °C with saturation humidity for 24 h or 48 h. After that, ten μL CCK‐8 solution (Dojindo, Japan) was added to 96‐well plates for 2 h. Finally, the absorbance at 450 nm was measured using a microplate reader (Bio-Tek, USA). Each experiment was repeated three times, and each experiment included three replicates.

The tunel assay kit (Servicebio, China) was performed to measure apoptosis. DMSCs were pretreated with 0 ng/ml, 3 ng/ml, 10 ng/ml, 30 ng/ml, and 100 ng/ml for 24 and 48 h, respectively, and then exposed to 6-OHDA for 2 h. Cells were then fixed with 4% paraformaldehyde and subjected to the TUNEL reaction according to the manufacturer's protocol. Cells were then fixed in 4% paraformaldehyde and subjected to TUNEL reaction according to the manufacturer's protocol. Tunel positive cells were photographed and analyzed by laser scanning confocal microscopy (Nikon, Japan). The average values of triplicate measurements were used as each activity value.

### Isolate CD4^+^T cells from peripheral blood

Peripheral blood was drawn from healthy volunteers after informed consent. Lymphocyte separation medium was used to isolate lymphocytes and peripheral blood mononuclear cells ex vivo. Purified CD4^+^ T cells were separated from PBMCs by negative isolation using the magnetic-activated cell sorting technology (Miltenyi Biotec, USA).

### FSH on cytokines levels of DMSCs

DMSCs were seeded at a density of approximately 5 × 10^4^ cells/well in a 24-well cell culture plate with DMEM containing 10% FBS. After four hours of incubation (logarithmic growth phase of bacteria), mitomycin C (Sigma, German) was added to a final concentration of 0.3 mg/mL, and incubation was continued for 12 h. Following this, the cells were divided into six groups, FSH + DMSC + CD4^+^T, FSH + CD4^+^T, FSH + DMSC, DMSC + CD4^+^T, DMSCs and CD4^+^T. Different concentrations of FSH (3, 10, 30, 100 ng/ml) were added to FSH groups. Following incubation at 37 °C for 24 or 48 h, levels of Interferon-γ (IFN-γ), interleukin-2 (IL-2), interleukin-4 (IL-4), interleukin-6 (IL-6)、interleukin-10(IL-10) and Tumor necrosis factor-α (TNF-α) (Millipore Corporation, USA) were measured using the Luminex 200™ analyzer (Immune Monitoring Core, USA). Each experiment was repeated three times, and each experiment included three replicates. The average values of triplicate measurements were used as each activity value.

### Western blot analysis

Western blot analysis was used to follicle-stimulating hormone receptor (FSHR), interleukin-6 (IL-6), determine myeloid differentiation factor 88(MyD88), Tumor necrosis factor-α (TNF-α), interleukin-2 (IL-2), interleukin-10 (IL-10) (Abcam, U.K.) expression in DMSCs. In brief, protein samples were separated using 10% SDS-PAGE and transferred onto PVDF membranes. Membranes were blocked with 5% nonfat milk in PBS containing 0.1% Tween-20 (Sigma, St Louis, USA) at 4 °C overnight under gentle rocking and incubated with primary antibodies at 4 °C. Membranes were washed three times and probed with goat anti-human secondary antibody (1:3000; Dawen, Hangzhou, China) for 2 h at room temperature. Immunoblots were visualized using an ECL detection kit (Amersham Biosciences, Pittsburgh, USA) and exposed to X-ray films. Each experiment was repeated three times, and each experiment included three replicates. The average values of triplicate measurements were used as each activity value.

### Statistics

Statistical analysis was performed with SPSS 19.0 software. The measurement data were subject to the normal distribution, expressed as the mean ± standard deviation. One-way ANOVA with Tukey's post-hoc test was used to compare groups, and *P* < 0.05 was considered a statistically significant difference. The statistical charts were designed with GraphPad Prism 7.0.

## Results

### Morphological characteristics of DMSCs

DMSCs were successfully isolated from first-trimester decidua, containing many epithelioid cells (Fig S1). After two or three passages, the epithelioid cells were significantly reduced, and the rest 95% of cells were fibroblasts, spindle-like, with nuclei centred and abundant cytoplasm (Fig S1).

### Analysis by flow cytometry

Flow cytometry with antibodies against specific markers for mesenchymal cells was applied to analyze the surface markers expressed by the cultured DMSCs. According to flow cytometry, DMSCs showed high-intensity expression of CD29 (C)、CD90 (D)、CD73 (E) and CD146 (G) in 42.9%-74.7% of the cells analyzed, but weak expression of CD45 (F)、CD3 (A)、CD19 (B)、HLA DR (H) and CD34 (I) in 2.2%-5.1% of the cells analyzed (Fig S2).

### Differentiation of DMSCs

After 21 days of osteogenic induction and culture of the fourth-generation DMSCs, it can be detected that the bone nodules were stained into red crystals by staining with Alizarin Red (Fig S3A). After 18 days of adipogenic induction and culture, the fourth-generation DMSCs were stained with oil red, and many fat droplets stained orange-red appeared in the cells (Fig S3B).

### Growth curve of DMSCs

The isolated DMSCs grew well and proliferated actively. The initial cell number was 1 × 10^4^/well, and the exponential growth phase doubling speed was about 28 h, stabilizing after 12 days (Fig S3C).

### Expression of FSHR mRNA and protein in DMSCs

**Fig. 1:**
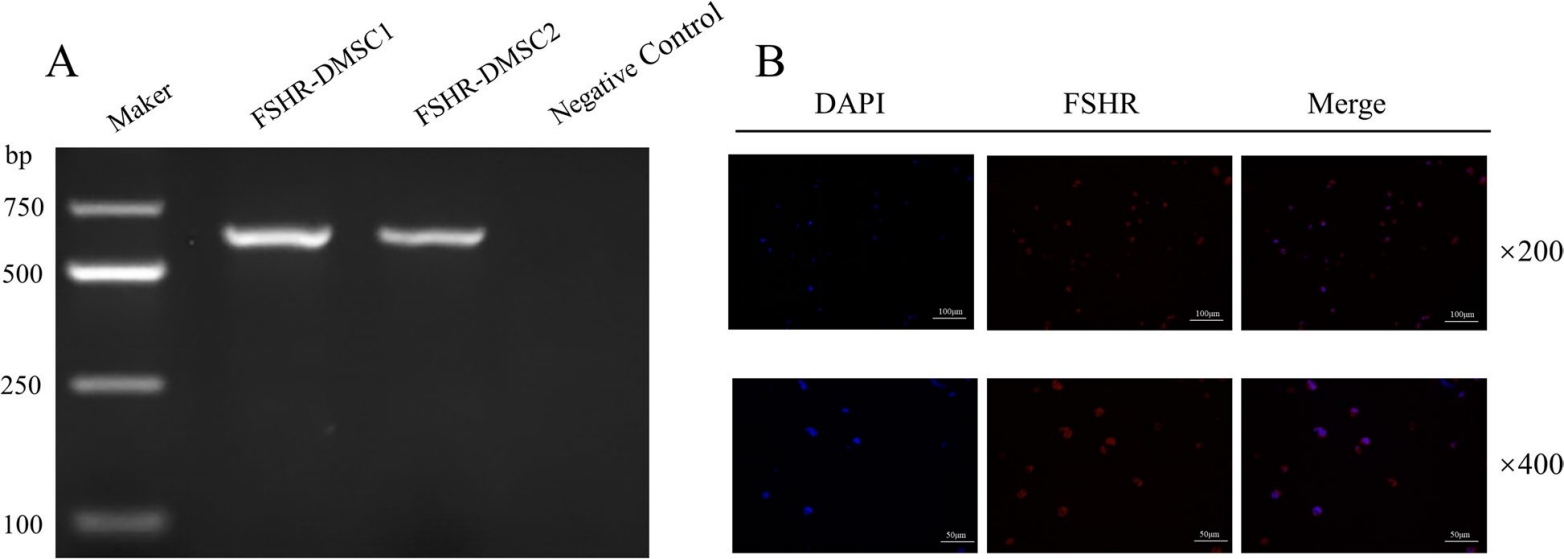
Immunofluorescence and RT-PCR identification of FSHR in DMSCs. **A** RT-PCR identification of FSHR using cDNA of FSHR transformants. Maker: DNA marker; FSHR-DMSC1 and FSHR-DMSC1: FSHR RNA expression in DMSC isolated from two decidua tissues. **B** Immunofluorescence and confocal microscopy assays showing FSHR protein expression in DMSCs using anti-EhPC4 and FIT-C conjugated secondary antibodies. Phase contrast, DAPI stain, green channel (FIT-C), and merge are indicated. Bottom panels, single cell (200X and 400X magnification). Green = FSHR, blue = DAPI

The results of RT-PCR revealed that FSHR mRNA was expressed in DMSCs (Fig. 1A). Similarly, immunofluorescence results revealed the presence of FSHR protein in DMSCs (Fig. 1B).

### The effect of FSH on the proliferation and apoptosis of DMSCs

**Fig. 2:**
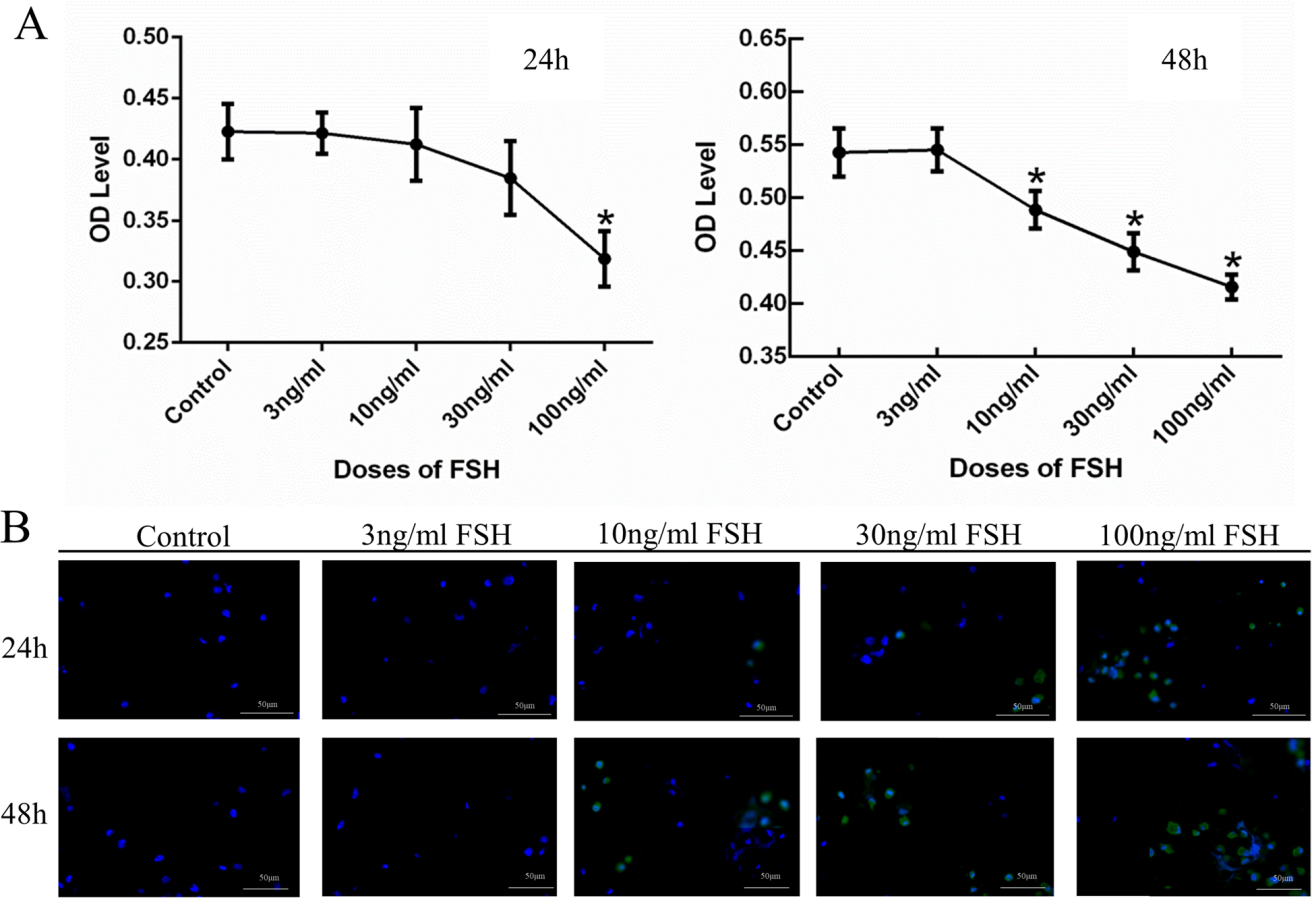
The effect of FSH on the proliferation and apoptosis of DMSCs. **A** Different concentrations of FSH acted on DMSCs for 24 h and 48 h by CCK-8 method. It had an inhibitory effect on the proliferation of DMSCs at 100 ng/ml for 24 h (*P* < 0.05). It had an inhibitory effect on the proliferation of DMSCs at 10, 30, and 100 ng/ml for 48 h (*P* < 0.05). The results shown are means ± SEM. **B** Terminal deoxynucleotidyl transferase dUTP nick end labelling (TUNEL) assay to detect apoptotic cell death. Representative microscopic images of TUNEL-positive cells in DMSCs treated with different doses of FSH at 24 h and 48 h. The results showed that high doses and prolonged FSH contributed to the apoptosis of DMSCs. Red = tunnel; blue = DAPI. scale bar = 50 μm (400X magnification)

By CCK-8 detection, the results showed that when FSH acted on DMSCs for 24 h, it had no significant impact on the proliferation of DMSCs at 3, 10, and 30 ng/ml (*P* > 0.05), and it had an inhibitory effect on the proliferation of DMSCs at 100 ng/ml (*P* < 0.05) (Fig. 2A). When the concentration of FSH acts on DMSCs for 48 h, it had no significant effect on the proliferation of DMSCs at 3 ng/ml (*P* > 0.05). It had an inhibitory effect on the proliferation of DMSCs at concentrations of 10, 30, and 100 ng/ml (*P* < 0.05) (Fig. 2A). The immunofluorescence results indicated that DMSCs exhibited a significant increase in Tunel-positive cells in response to high doses of FSH (100 ng/ml). The results were higher at 48 h of effect than at 24 h (Fig. 2B).

### The effect of FSH on cytokine secretion of DMSCs

**Fig. 3:**
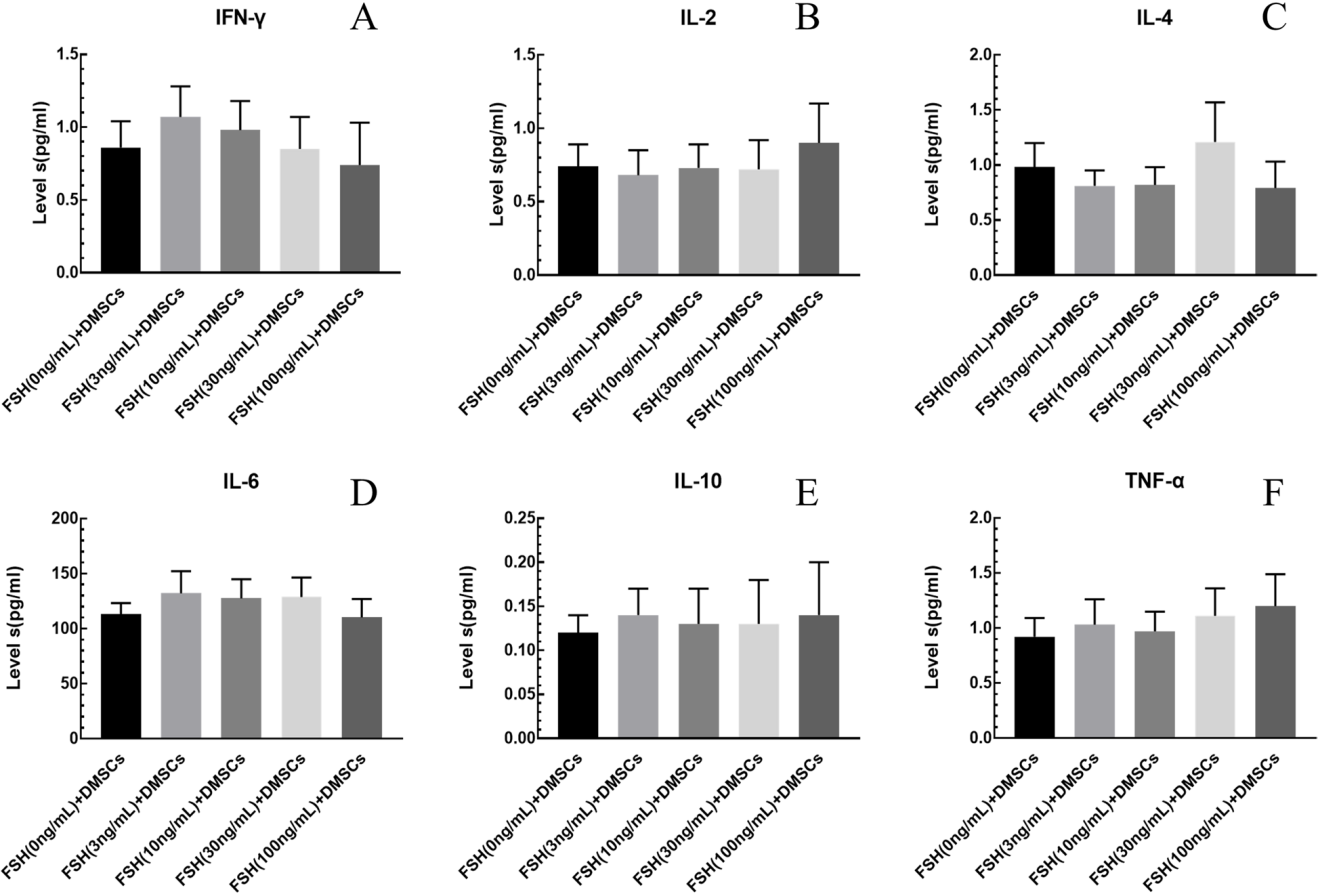
The effect of FSH on cytokine secretion of DMSCs. There was no significant difference with various cytokines levels (IFN-γ, IL-2, IL-4, IL-6, IL-10, TNF-α) compared with the control group (FSH (0 ng/mL) + DMSCs), (*P* > 0.05). The results shown are means ± SEM

After different concentrations of FSH (3, 10, 30, 100 ng/ml) on DMSCs for 48 h, there was no significant difference with various cytokines levels (IFN-γ, IL-2, IL-4, IL-6, IL-10, TNF-α) compared with the control group (FSH (0 ng/mL) + DMSCs), (*P* > 0.05) (Fig. 3).

### The effect of DMSCs on lymphocyte cytokine secretion

**Fig. 4:**
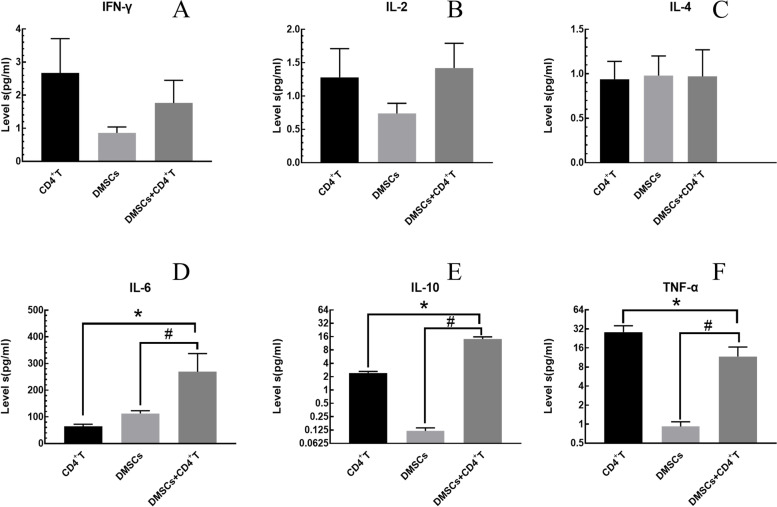
The effect of DMSCs on lymphocyte cytokine secretion. Compared with the CD4 + T group, the levels of IL-6 and IL-10 in the DMSCs + CD4 + T group were significantly increased (*P* < 0.05), and the level of TNF-α was significantly increased (*P* < 0.05). Compared with the DMSCs group, IL-6, IL-10, and TNF-α in the DMSCs + CD4 + T group were significantly increased (*P* < 0.05). The results shown are means ± SEM

Compared with the CD4 + T group, the levels of IL-6 and IL-10 in the DMSCs + CD4^+^T group were significantly increased (*P* < 0.05), and the level of TNF-α was significantly increased (*P* < 0.05). Compared with the DMSCs group, the levels of IL-6, IL-10, and TNF-α in the DMSCs + CD4^+^T group were significantly increased (*P* < 0.05) (Fig. 4).

### The effect of FSH on cytokine secretion of DMSCs + CD^+^T Co-Culture System

**Fig. 5:**
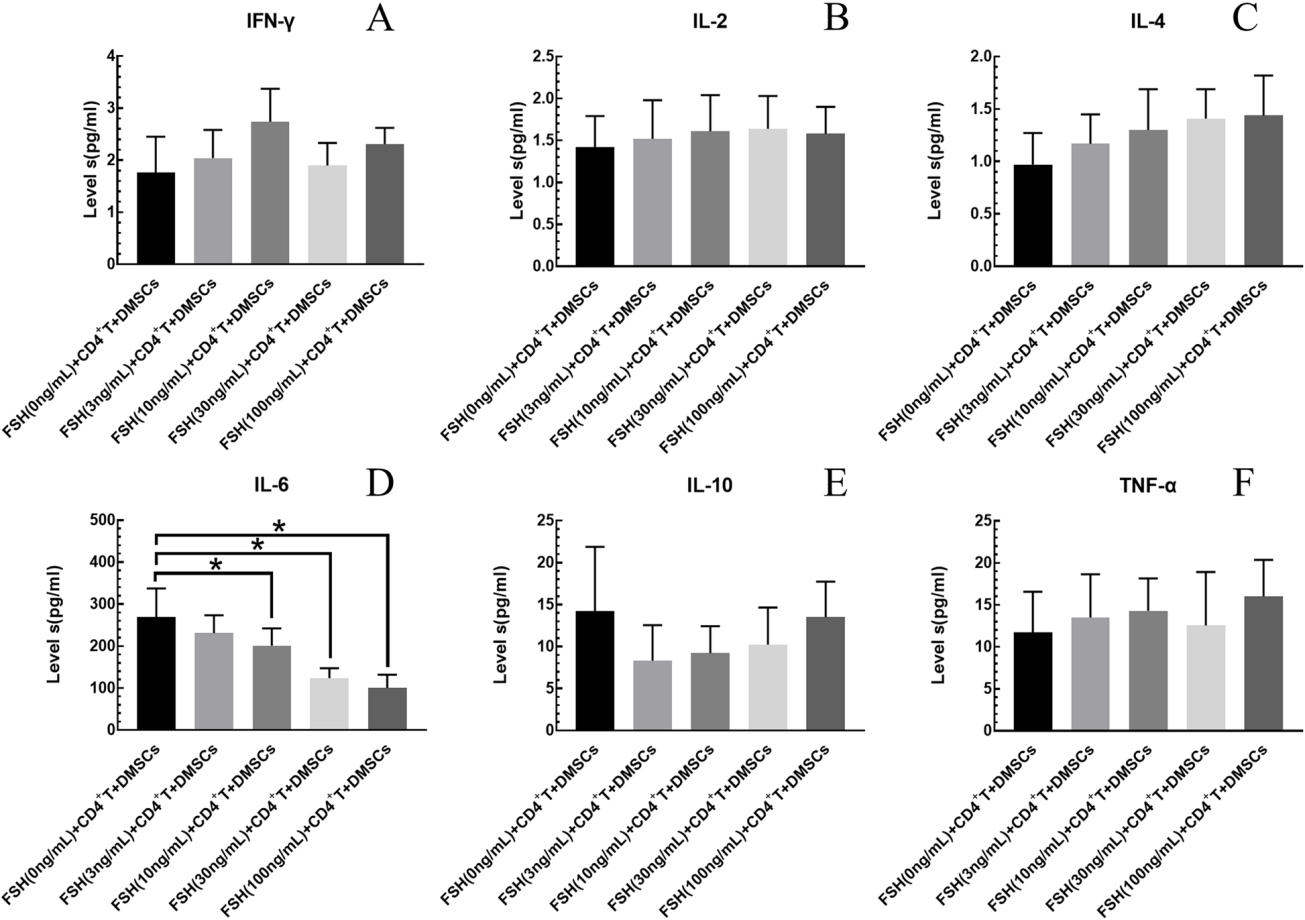
The effect of FSH on cytokine secretion of DMSCs + CD + T Co-Culture System. Compared with the control group (FSH (0 ng/mL) + CD4 + T + DMSCs), the FSH concentration was 10, 30, and 100 ng/ml, IL-6 levels were significantly reduced (*P* < 0.05). The results shown are means ± SEM

Different concentrations of FSH (3, 10, 30, 100 ng/ml) were added to the CD4^+^T + DMSCs co-culture system for 48 h. Compared with the control group (FSH (0 ng/mL) + CD4^+^T + DMSCs), the FSH concentration was 10, 30, and 100 ng/ml, IL-6 levels were significantly reduced (*P* < 0.05) (Fig. 5).

### The effect of FSH on protein expression of DMSCs + CD4^+^T co-culture system

**Fig. 6:**
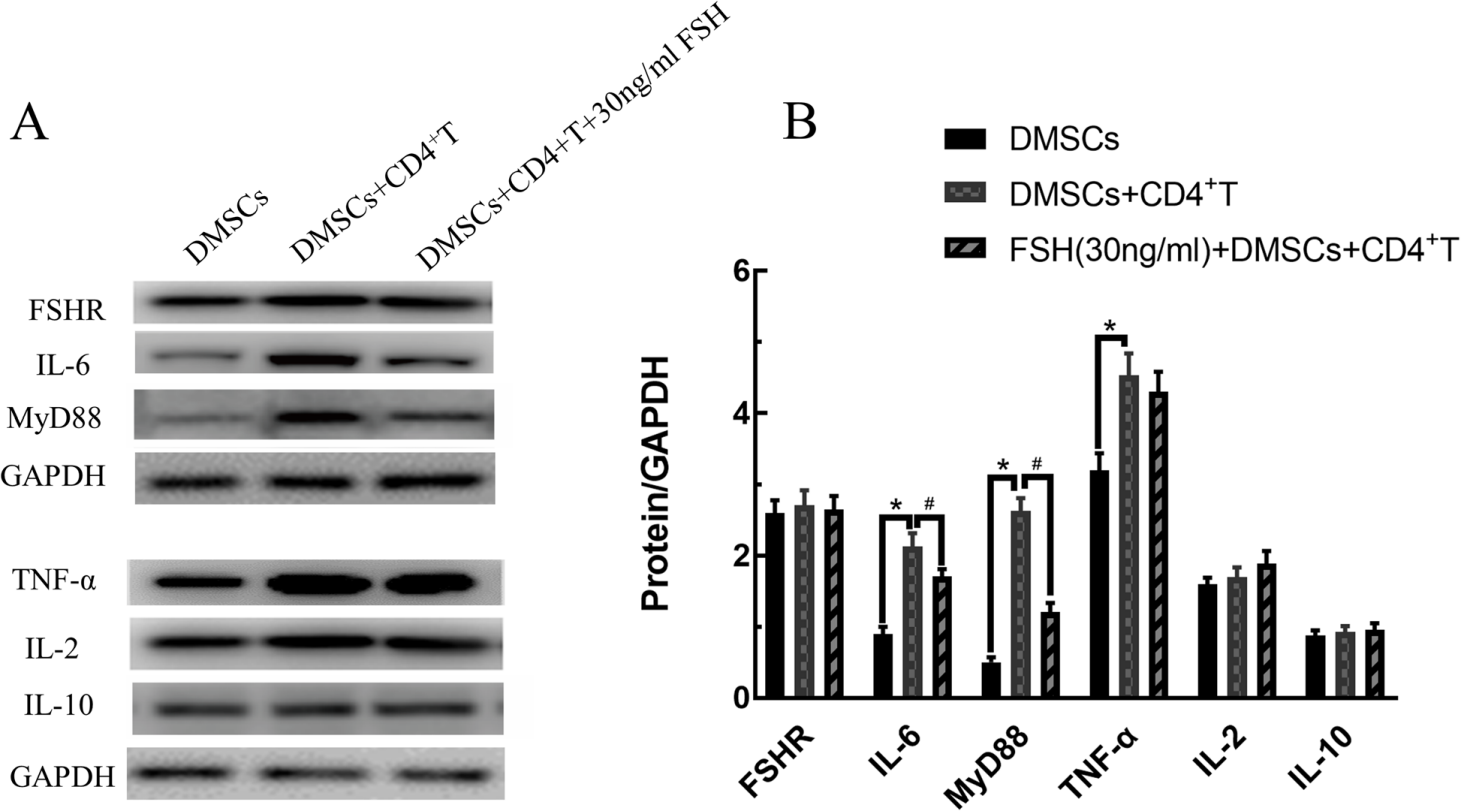
The effect of FSH on protein expression of DMSCs + CD4 + T Co-Culture System. IL-6, MyD88 protein expression was remarkably decreased in the experimental group (FSH (30 ng/mL) + CD4 + T + DMSCs) compared with control group (DMSCs + CD4 + T) (*P* < 0.05). IL-6, MyD88, TNF-α protein expression was remarkably increased in control group (DMSCs + CD4 + T) compared with Blank group (DMSCs alone) (*P* < 0.05). Results shown are means ± SEM

According to the above results, 30 ng/ml of FSH acted on the co-culture system for 48 h in the experimental group. IL-6, MyD88 protein expression was remarkably decreased in the experimental group (FSH (30 ng/mL) + CD4^+^T + DMSCs) compared with control group (DMSCs + CD4^+^T) (*P* < 0.05). IL-6, MyD88, TNF-α protein expression was remarkably increased in control group (DMSCs + CD4 + T) compared with Blank group (DMSCs alone) (Fig. 6) (*P* < 0.05).

## Discussion

Decidua is a special tissue formed by the invasion of trophoblast cells in the maternal endometrium[16]. It includes the maternal–fetal interface with the trophoblast cells on the offspring surface. It provides a suitable growth environment and immune environment for the fetus and plays a role in maintaining early pregnancy. Decidual mesenchymal stem cells (DMSCs) are an essential part of decidual tissue and may be associated with term spontaneous labour [8]. DMSCs have similar properties and lower immunogenicity than BMSCs. In recent years, they have attracted the attention of many scholars and have significant clinical application prospects in regenerative medicine, wound repair, and hematopoietic stem cell transplantation [17].

Dimitrov’s method [18] was used to isolate DMSCs, and it was found that the isolated primary DMSCs had a lot of red blood cells, which affected cell viability. The fourth-generation fibroblast-like cells cannot reach more than 95% of total cells by Dimitrov's method. We improved Dimitrov's method by adding the steps of rupturing red blood cells with 0.84% ​​NH_4_CL and filtering with a 200-mesh filter, effectively purifying the primary cells. The decidual mesenchymal stem cells isolated have good cell viability, fibroblast-like spiral growth adherent to the wall, and differentiation ability of bone formation and adipogenesis. It was found that our cells separated from decidua meet the minimum MSC standards established by ISCT (International Association for Cell Therapy) [2] and the standards set by the first International Working Group on Placental Stem Cells [19] through immunophenotype by flow cytometry. We found that the high expression of CD146 in the DMSCs isolated in this study (74.7%) was consistent with the results of Dimitrov [18] (Fig S2G). At the same time, neither bone marrow nor fat-derived MSCs had reported a positive phenotype, indicating that CD146 might be s a phenotype that could distinguish DMSCs from other sources of MSCs, but more studies should be confirmed. At the same time, studies have shown that DMSCs could also differentiate into endoderm-derived respiratory epithelial cells and lung cells, indicating that DMSCs have more substantial multi-directional differentiation potential [20]. The proliferation rate of DMSCs isolated in this study (exponential growth rate was about 28 h) was slightly higher than that of BM-MSCs (exponential growth rate was about 31 h) but somewhat lower than other related literature reports (exponential growth rate was about 21.3 h) [21] (Fig S3C). Many factors could change the proliferation rate of DMSCs [3], but hypoxia was the main focus of current research. Studies have shown that hypoxia could promote the proliferation of DMSCs, indicating that its growth rate was closely related to oxygen concentration, and this mechanism might be associated with the up-regulation of BCL-2 levels [3].

The rapid development of allogeneic hematopoietic stem cell transplantation has a broad role in treating hematologic disorders, such as leukaemia, lymphoma, and aplastic anaemia. However, this type of stem cell transplantation complication can not be ignored, especially a severe complication for graft-versus-host response (GVHD). High mortality and difficult-to-treat characteristics quickly make it a medical problem [22]. Le Blanc et al. use allogeneic MSCs to treat acute and hormone ineffective GVHD and achieve a good treatment effect [23]. Kim N et al. find that MSC has an immunosuppressive effect of decreasing GVHD incidence. Besides, other studies have found that MSCs can inhibit the proliferation of various immune cells such as T cells, NK cells, and DC in vitro, and immune cells constitute a stable microenvironment for maintaining pregnancy [4]. The above studies indicate that DMSCs participate in the immune tolerance of the mother-fetal interface. Further research on DMSCs could improve the theory of reproductive immunity and benefit patients with infertility.

The immune regulation of DMSCs is mainly through the secretion of immune regulatory factors and directly affects the number of immune cells such as T cells, NK cells, and DCs. There are many cytokines in the microenvironment of the maternal–fetal interface, such as IFN-γ, IL-2, IL-4, IL-6, IL-10, TNF-α, which influence the maternal immune tolerance [24]. MSC can secrete IL-10, TGF-β, IDO, and PGE2, thereby inhibiting the proliferation of T cells and exerting immunosuppressive functions [6]. Human leukocyte antigen-G (HLA-G) is an essential factor that mediates immune tolerance. DMSCs have had low expression of HLA-G for a long time [25]. When stimulated by progesterone and some anti-inflammatory factors such as IL-10, the expression level of HLA-G increased significantly, showing that DMSCs play an essential role in maintaining pregnancy immune stability [25]. Progesterone-induced blocking factor (PIBF) is a vital activity composition in the stability of maternal–fetal immune tolerance. It can inhibit Th1 immune response and Treg cell proliferation. PIBF is continuously released in DMSCs, and some sex hormones such as progesterone will regulate its expression [25, 26].

Follicle-stimulating hormone (FSH) is released from the anterior pituitary gland in the hypothalamus. For women, it can promote the proliferation and differentiation of follicular granulosa cells and accelerate the growth and development of ovaries. For men, it can act on the seminiferous tubules of the testes and participate in sperm production [27]. The effect of FSH on target organs is mainly mediated by FSH receptor (FSHR) [28, 29]. Previous studies propose that FSH is only expressed in female ovarian granulosa cells and male testicular Sertoli cells. However, recent studies have shown that FSHR is not expressed only in the gonads, find that FSHR is expressed in the placenta, liver, fat, muscle, and skin tissue expression [30], and also exists on BMSCs [10]. The high and abundant FSHR expression at protein levels by immunohistochemistry or immunofluorescence is not associated with clear FSHR mRNA amplification [31], the identification of FSHR requires the combination of mRNA and protein results. Recent study indicate that FSH can mobilizes VSELs and HSPCs into peripheral blood [32, 33], indicate FSH also has an association with hematopoietic function.

It has been statistically shown that high FSH levels are not conducive to the maintenance of pregnancy [14]. Moreover, we found that FSHR receptors existed in DMSCs. We speculated that the FSH/FSHR pathway was a meaningful way to mediate the immune regulation function of DMSCs, but no function-related studies of them were found.

Our research found FSH can inhibit the proliferation of DMSCs in vitro, and higher concentrations have a better inhibitory effect on the proliferation of DMSCs (*P* < 0.05) (Fig. 2A). The inhibitory effect was dose-dependent. The higher the concentration, the stronger the inhibitory effect. Moreover, the effect of 48 h was more evident than 24 h. It indicated that FSH could directly affect the proliferation ability of DMSCs in a dose- and time-dependent manner. This result was supported by the tunel assay (Fig. 2B), showing that high concentrations of FSH promote apoptosis of DMSCs. However, the mechanism needed to be further investigated.

We had established a co-culture system of DMSCs and CD4^+^ T lymphocytes, pretreated DMSCs with Mitomycin C, added different physiological concentrations of FSH (3, 10, 30, 100 ng/ml) to the co-culture system, and observed the changes in cytokines levels. In the DMSCs + CD4^+^T co-culture system, IL-6 and IL-10 levels were significantly increased than those in the CD4^+^T culture group, while the secretion levels of TNF-α were significantly reduced. The results were consistent with Aggarwal, S [6]. TNF-α was both an inflammatory factor and a Th1 cytokine. IL-10 was an immunosuppressive factor and a Th2 cytokine, showing that DMSCs had an immunosuppressive effect on pregnancy immunity to maintain pregnancy by shifting Th1/Th2 balance towards Th2. IL-6 had a wide range of biological effects. It could stimulate the growth of various cells and promote the synthesis of plasma albumin. Besides, IL-6 was mainly used as Th2 cytokines to mediate fluid immunity to maintain allogeneic immune tolerance During pregnancy [34]. DMSCs and lymphocytes could secrete IL-6 individually [35]. Our study found that DMSCs could promote CD4^+^T cells to secrete more IL-6 in the DMSCs + CD4^+^T co-culture system.

Different physiological concentrations of FSH (3, 10, 30, 100 ng/ml) added on DMSCs pretreated with mitomycin C, compared with the control group (FSH(0 ng/ml) + DMSCS), the cytokines (IFN-γ, IL-2, IL-4, IL-6, IL-10, TNF-α) were no significant change (*P* > 0.05) (Fig. 3). The reason might be that mitomycin C prevents cell proliferation and affect the secretion of cytokines, or maybe FSH did not affect cytokines levels in DMSCs alone. When different concentrations of FSH acted on the DMSCs + CD4^+^T co-culture system, higher concentrations of FSH (30, 100 ng/ml) would significantly decrease the secretion level of IL-6 (Fig. 5) indicating that the immune regulation of FSH on DMSCs requires the support of CD4^+^T lymphocytes. Lower concentrations of FSH (3, 10 ng/ml) had no effect. FSH directly affected various gonadal cells and stem cells [36]. Still, no studies had shown that there was FSHR on the surface of lymphocytes. We measured the effect of FSH on cytokine secretion of Co-Culture System, which did not prove whether FSH can act on CD4^+^ T cells. Theoretically, FSH could not act directly on lymphocytes, but this still needed to be demonstrated experimentally. This limitation of our study made our results more conservative, and we would add relevant experiments in future studies. IL-6 protein expression was decreased in the experimental group (FSH (30 ng/mL) + CD4^+^T + DMSCs) compared with control group (DMSCs + CD4^+^T), which was consistent with the results tested by Luminex 200™ System. Western blot results indicated DMSCs exist as follicle-stimulating hormone receptors (FSHR). MyD88 protein expression decreased simultaneously with IL-6 (Fig. 6), revealing that the effect of FSH in co-cultivation was associated with MyD88.

Toll-like receptors (TLR) are the primary receptors for the innate immune system to recognize pathogen-related molecular patterns. The signalling pathways initiated by them can also regulate adaptive immune responses. MyD88 is an essential transduction protein in this signal pathway [37]. IL-6 was almost exclusively TLR4 dependent and relied on MyD88 and TRIF adaptor cooperation. TLR4 initially recruits TIRAP at the plasma membrane. Subsequently, it facilitates the recruitment of MyD88 to trigger the initial activation of NF-κB, which is necessary for the induction of IL-6 levels via TLR4 signalling [38].

## Conclusions

In summary, DMSCs had immunosuppressive effects, could enhance the secretion level of IL-6, and IL-10 reduced the secretion level of TNF-α and made the Th1/Th2 balance shift to Th2 to maintain pregnancy immune tolerance. FSH/FSHR could inhibit the proliferation and promote apoptosis of DMSCs. Besides, FSH/FSHR could negatively regulate the immunosuppressive function of DMSCs by reducing secretion of IL-6 levels through MyD88 pathways. Still, upstream and downstream signalling pathways required further validation. There was no relevant report on the regulatory effect of FSH on immune function of DMSCs. We expected this research could improve development of the pathogenesis research and treatment of abnormal pregnancy such as threatened abortion and missed abortion.

## Supplementary Information

**Additional file 1:** **Figure S1.** Morphological characteristics of DMSCs. (A) human decidual tissue. (B) the first generation DMSCs isolated from first trimester decidua contained many epithelioid cells. (C) The fourth-generation decidual mesenchymal stem cells with fibroblasts are spindle-like, nuclei centred, and abundant in cytoplasm. (D) Homogenous monolayer of DMSCs. Wright-Giemsa stain. Bar = 200μm.

**Additional file 2:** **Figure S2.** Phenotypic analysis. According to flow cytometry, DMSCs showed high-intensity expression of CD29 (C), CD90 (D), CD73 (E), and CD146 (G) in 42.9%-74.7% of the cells analyzed, but weak expression of CD45 (F), CD3 (A), CD19 (B), HLA DR (H) and CD34 (I) in 2.2%-5.1% of the cells analyzed.

**Additional file 3:** **Figure S3.** DMSCs could be induced to differentiate as osteogenic cells or adipogenic cells. (A) osteogenic cells. Alizarin red stain. (B) adipogenic cells. Oil red stain. Bar = 100μm. (C) Growth curve of DMSCs. The initial cell number was 1×104/well, and the exponential growth phase doubling speed was about 28h, stabilizing after 12 days. The results shown are means ± SEM.

## Data Availability

Not applicable.

## Abbreviations

FSH: Follicle Stimulating Hormone
DMSCs: Decidual Mesenchymal Stem Cells
MyD88: Myeloid Differentiation Factor 88
IL-6: Interleukin-6
IVF: In vitro fertilization
MSCs: Mesenchymal stem cells
FSHR: follicle-stimulating hormone receptors
IFN-γ: Interferon-γ
IL-2: Interleukin-2
IL-4: Interleukin-4
IL-10: Interleukin-10
TNF-α: Tumor necrosis factor-α
GVHD: Graft-versus-host response
PIBF: Progesterone induced blocking factor
TLR: Toll-like receptors
NF-κB: Nuclear Factor-kappa B

## References

[1] Haydont V, Neiveyans V, Perez P, Busson E, Lataillade J, Asselineau D (2020). Fibroblasts from the Human Skin Dermo-Hypodermal Junction are Distinct from Dermal Papillary and Reticular Fibroblasts and from Mesenchymal Stem Cells and Exhibit a Specific Molecular Profile Related to Extracellular Matrix Organization and Modeling. Cells.

[2] Karpov AA, Drahova AV, Buslova DV, Ivkin DY, Moiseeva OM, Galagudza MM (2015). Modification of Mesenchymal Stem Cells as a Way To Improve the Effectiveness of Cell Therapy of Ischemic Myocardial Injury. Ross Fiziol Zh Im I M Sechenova.

[3] Huang YC, Yang ZM, Chen XH, Tan MY, Wang J, Li XQ (2009). Isolation of mesenchymal stem cells from human placental decidua basalis and resistance to hypoxia and serum deprivation. Stem Cell Rev Rep.

[4] Roelen DL, van der Mast BJ, in’t Anker PS, Kleijburg C, Eikmans M, van Beelen E (2009). Differential immunomodulatory effects of fetal versus maternal multipotent stromal cells. Hum Immunol.

[5] Murata H, Tanaka S, Okada H (2021). Immune Tolerance of the Human Decidua. J Clin Med.

[6] Aggarwal S, Pittenger MF (2005). Human mesenchymal stem cells modulate allogeneic immune cell responses. Blood.

[7] Choi YS, Park YB, Ha CW, Kim JA, Heo JC, Han WJ (2017). Different characteristics of mesenchymal stem cells isolated from different layers of full term placenta. PLoS One.

[8] Wijaya JC, Khanabdali R, Georgiou HM, Kokkinos MI, James PF, Brennecke SP (2020). Functional changes in decidual mesenchymal stem/stromal cells are associated with spontaneous onset of labour. Mol Hum Reprod.

[9] Zhu XY1D, Sun XX (2003). Characters of cytokine expression at maternal-fetal interface in normal pregnancy and in spontaneous abortion mouse models. J Reprod Med.

[10] Sun L, Peng Y, Sharrow AC, Iqbal J, Zhang Z, Papachristou DJ (2006). FSH directly regulates bone mass. Cell..

[11] Balasch J, Creus M, Fabregues F, Carmona F, Casamitjana R, Ascaso C (1996). Inhibin, follicle-stimulating hormone, and age as predictors of ovarian response in in vitro fertilization cycles stimulated with gonadotropin-releasing hormone agonist-gonadotropin treatment. Am J Obstet Gynecol.

[12] Barnhart K, Osheroff J (1998). Follicle stimulating hormone as a predictor of fertility. Curr Opin Obstet Gynecol.

[13] Scott RT, Hofmann GE (1995). Prognostic assessment of ovarian reserve. Fertil Steril.

[14] Sl C (2011). Impact of elevated basal follicle-stimulating hormone on the quantity and quality of and embryos and pregnancy outcomes in young women. J South Med Univ.

[15] Thum MY, Kalu E, Abdalla H (2009). Elevated basal FSH and embryo quality: lessons from extended culture embryos: raised FSH and blastocyst quality. J Assist Reprod Genet.

[16] Tong J, Niu Y, Chen ZJ, Zhang C (2020). Comparison of the transcriptional profile in the decidua of early-onset and late-onset pre-eclampsia. J Obstet Gynaecol Res.

[17] Zhao J, Ding Y, He R, Huang K, Liu L, Jiang C (2020). Dose-effect relationship and molecular mechanism by which BMSC-derived exosomes promote peripheral nerve regeneration after crush injury. Stem Cell Res Ther.

[18] Dimitrov R, Kyurkchiev D, Timeva T, Yunakova M, Stamenova M, Shterev A (2010). First-trimester human decidua contains a population of mesenchymal stem cells. Fertil Steril.

[19] Parolini O, Alviano F, Bagnara GP, Bilic G, Buhring HJ, Evangelista M (2008). Concise review: isolation and characterization of cells from human term placenta: outcome of the first international Workshop on Placenta Derived Stem Cells. Stem Cells.

[20] Macias MI, Grande J, Moreno A, Dominguez I, Bornstein R, Flores AI (2010). Isolation and characterization of true mesenchymal stem cells derived from human term decidua capable of multilineage differentiation into all 3 embryonic layers. Am J Obstet Gynecol.

[21] Chen CP, Lee MY, Huang JP, Aplin JD, Wu YH, Hu CS (2008). Trafficking of multipotent mesenchymal stromal cells from maternal circulation through the placenta involves vascular endothelial growth factor receptor-1 and integrins. Stem Cells.

[22] Rashidi A, Khoruts A, Weisdorf DJ (2017). Infection Followed by Graft-versus-Host Disease: Pathogenic Role of Antibiotics. Biol Blood Marrow Transplant.

[23] Le Blanc K, Rasmusson I, Sundberg B, Gotherstrom C, Hassan M, Uzunel M (2004). Treatment of severe acute graft-versus-host disease with third party haploidentical mesenchymal stem cells. Lancet.

[24] Piccinni MP, Lombardelli L, Logiodice F, Kullolli O, Parronchi P, Romagnani S (2016). How pregnancy can affect autoimmune diseases progression?. Clin Mol Allergy.

[25] Blanco O, Tirado I, Munoz-Fernandez R, Abadia-Molina AC, Garcia-Pacheco JM, Pena J (2008). Human decidual stromal cells express HLA-G: Effects of cytokines and decidualization. Hum Reprod.

[26] Hudic I, Fatusic Z (2009). Progesterone - induced blocking factor (PIBF) and Th(1)/Th(2) cytokine in women with threatened spontaneous abortion. J Perinat Med.

[27] Dias JA (1992). Progress and approaches in mapping the surfaces of human follicle-stimulating hormone: comparison with the other human pituitary glycoprotein hormones. Trends Endocrinol Metab.

[28] Bogerd J (2007). Ligand-selective determinants in gonadotropin receptors. Mol Cell Endocrinol.

[29] Braun T, Schofield PR, Sprengel R (1991). Amino-terminal leucine-rich repeats in gonadotropin receptors determine hormone selectivity. EMBO J.

[30] Tsitlakidis D, Katopodi T, Goulis DG, Papadimas I, Kritis A (2017). Association of follicle-stimulating hormone receptor single nucleotide polymorphisms with fertility in Greek men. J Endocrinol Invest.

[31] Chrusciel M, Ponikwicka-Tyszko D, Wolczynski S, Huhtaniemi I, Rahman NA (2019). Extragonadal FSHR Expression and Function-Is It Real?. Front Endocrinol (Lausanne).

[32] Abdelbaset-Ismail A, Suszynska M, Borkowska S, Adamiak M, Ratajczak J, Kucia M (2016). Human haematopoietic stem/progenitor cells express several functional sex hormone receptors. J Cell Mol Med.

[33] Zbucka-Kretowska M, Eljaszewicz A, Lipinska D, Grubczak K, Rusak M, Mrugacz G (2016). Effective Mobilization of Very Small Embryonic-Like Stem Cells and Hematopoietic Stem/Progenitor Cells but Not Endothelial Progenitor Cells by Follicle-Stimulating Hormone Therapy. Stem Cells Int.

[34] Kamali-Sarvestani E, Zolghadri J, Gharesi-Fard B, Sarvari J (2005). Cytokine gene polymorphisms and susceptibility to recurrent pregnancy loss in Iranian women. J Reprod Immunol..

[35] Raghupathy R, Makhseed M, Azizieh F, Omu A, Gupta M, Farhat R (2000). Cytokine production by maternal lymphocytes during normal human pregnancy and in unexplained recurrent spontaneous abortion. Hum Reprod.

[36] Bhartiya D, Patel H (2021). An overview of FSH-FSHR biology and explaining the existing conundrums. J Ovarian Res.

[37] Li C, Zienkiewicz J, Hawiger J (2005). Interactive sites in the MyD88 Toll/interleukin (IL) 1 receptor domain responsible for coupling to the IL1beta signaling pathway. J Biol Chem.

[38] Royer PJ, Rogers AJ, Wooldridge KG, Tighe P, Mahdavi J, Rittig MG (2013). Deciphering the contribution of human meningothelial cells to the inflammatory and antimicrobial response at the meninges. Infect Immun.

