# Supplementary figures and images for: Effect of human follicle-stimulating hormone on immunomodulatory function of decidual mesenchymal stem cells by reducing interleukin-6 levels
Source: J Ovarian Res. 2022 May 13;15:60. doi: 10.1186/s13048-022-00993-3 (PMC9102716; doi:10.1186/s13048-022-00993-3)

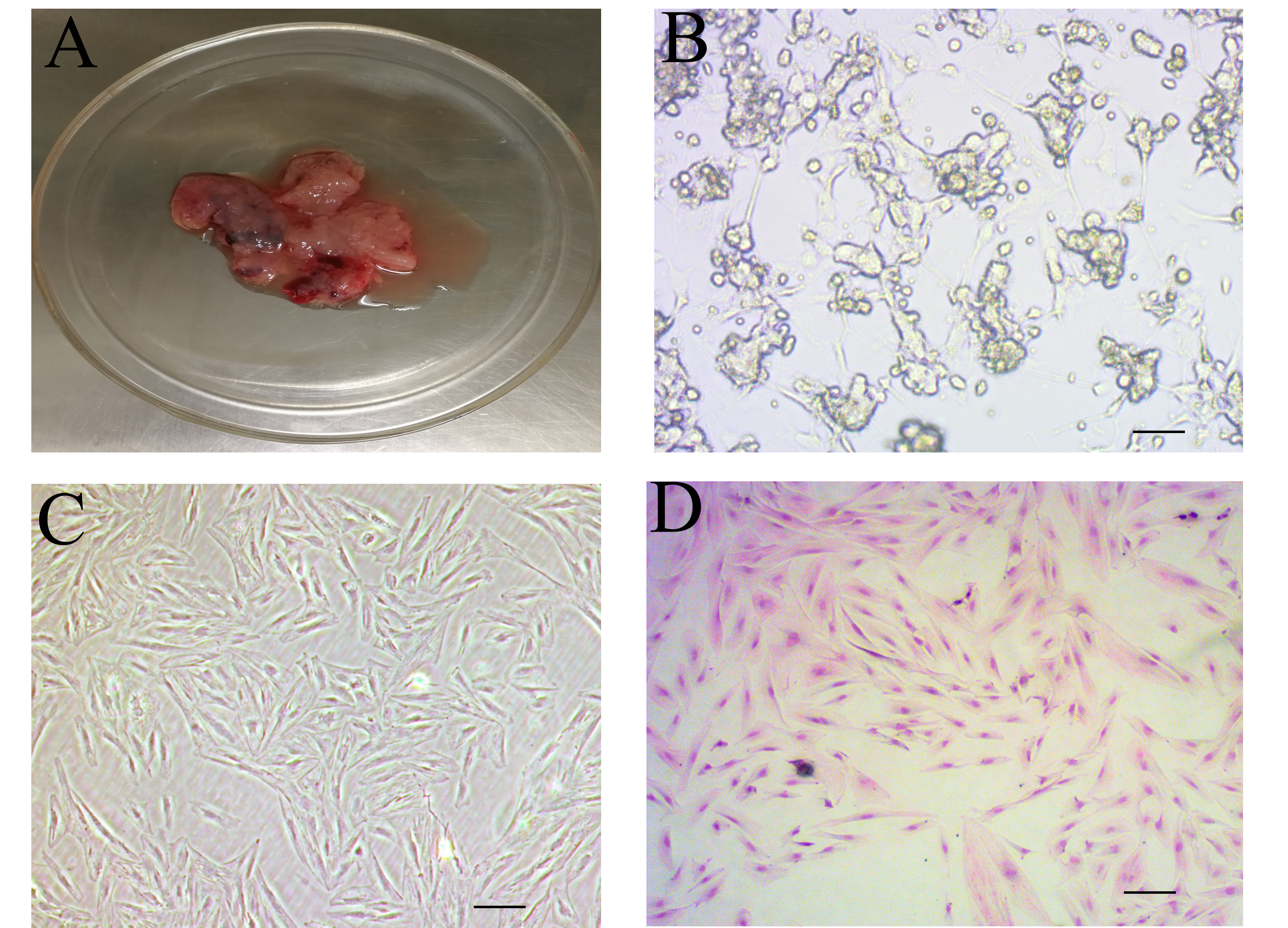

Supplement: Supplementary file 1 — Additional file 1: Figure S1. Morphological characteristics of DMSCs. (A) human decidual tissue. (B) the first generation DMSCs isolated from first trimester decidua contained many epithelioid cells. (C) The fourth-generation decidual mesenchymal stem cells with fibroblasts are spindle-like, nuclei centred, and abundant in cytoplasm. (D) Homogenous monolayer of DMSCs. Wright-Giemsa stain. Bar = 200μm. [file 13048_2022_993_MOESM1_ESM.tif]

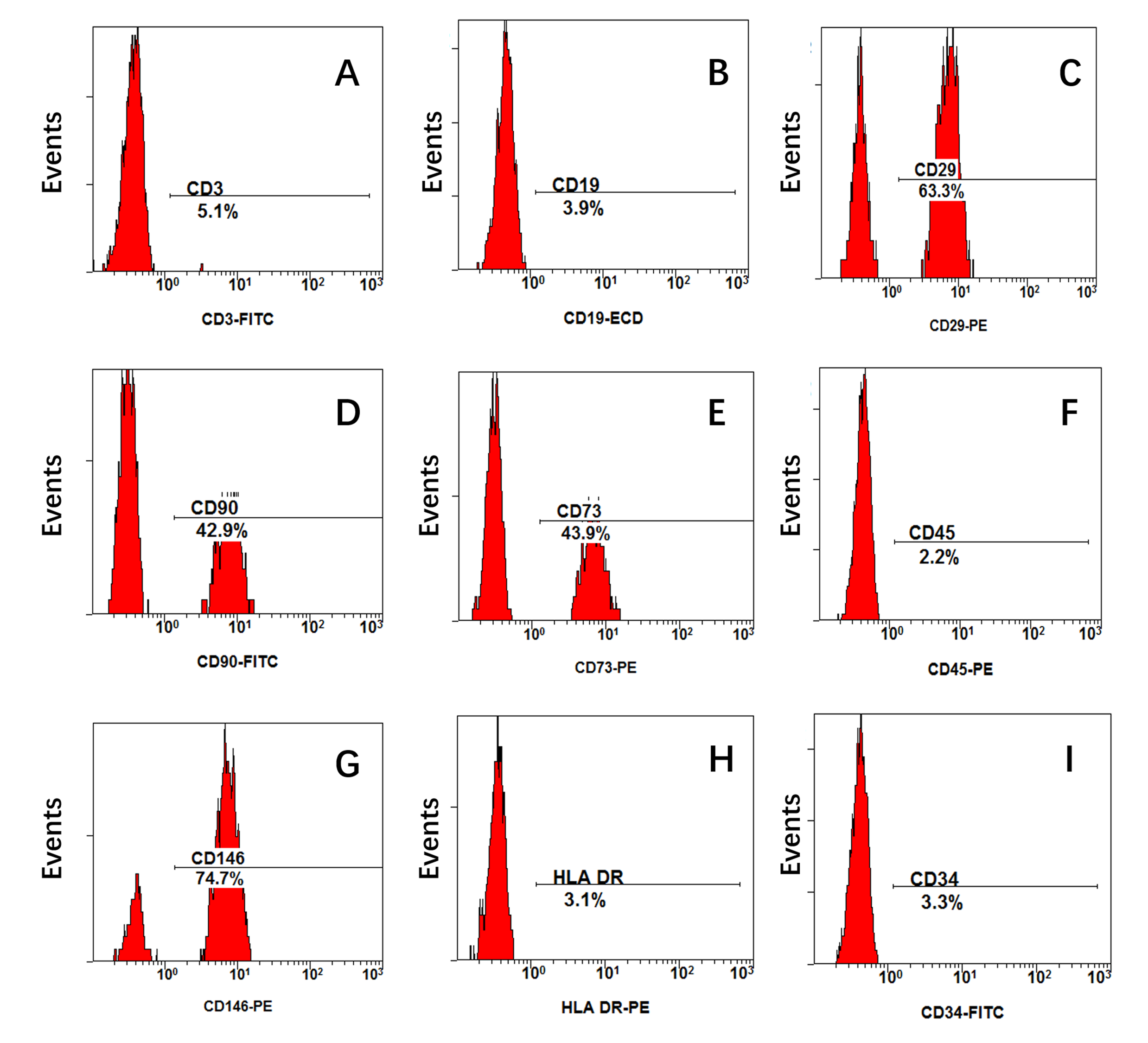

Supplement: Supplementary file 2 — Additional file 2: Figure S2. Phenotypic analysis. According to flow cytometry, DMSCs showed high-intensity expression of CD29 (C), CD90 (D), CD73 (E), and CD146 (G) in 42.9%-74.7% of the cells analyzed, but weak expression of CD45 (F), CD3 (A), CD19 (B), HLA DR (H) and CD34 (I) in 2.2%-5.1% of the cells analyzed. [file 13048_2022_993_MOESM2_ESM.tif]

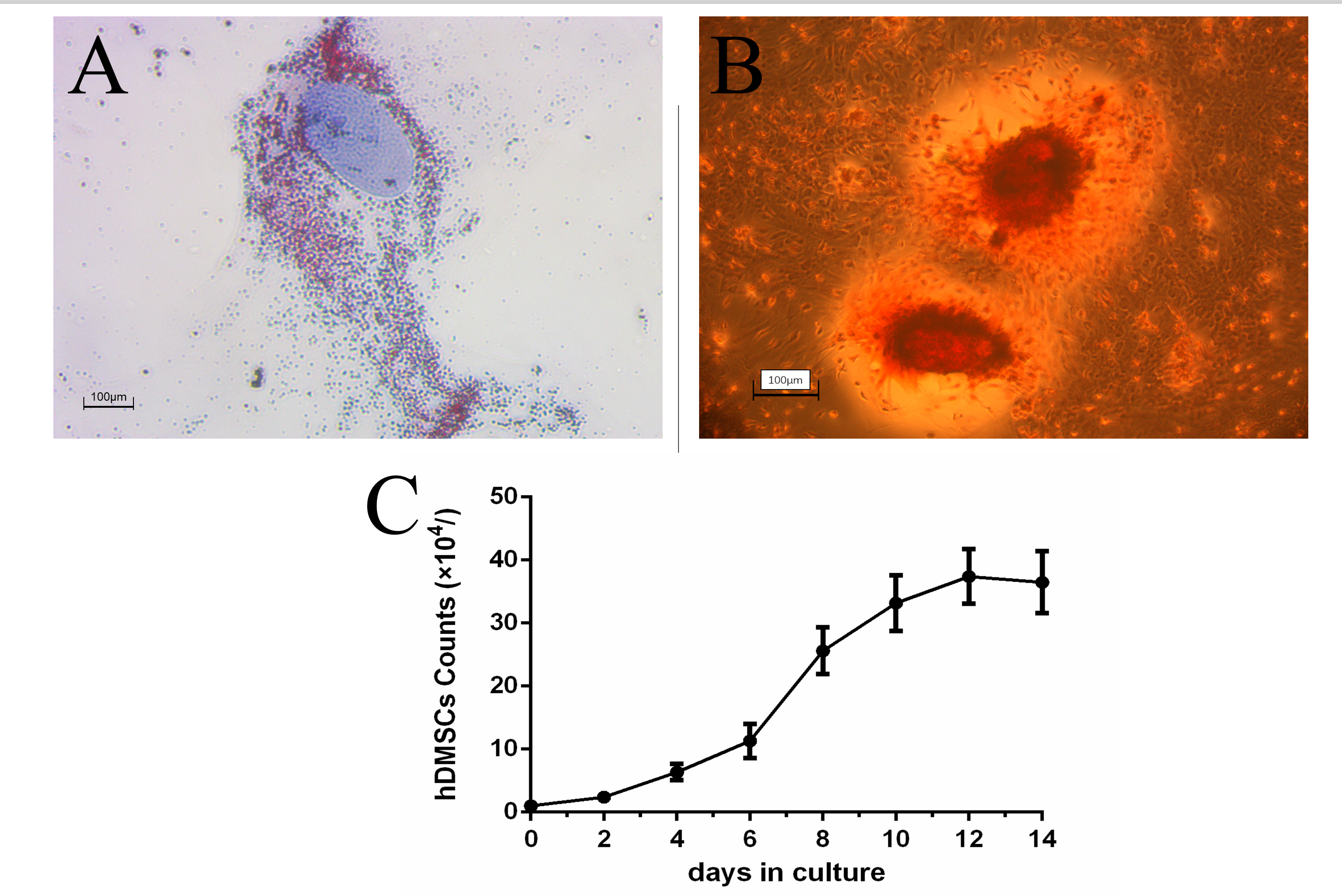

Supplement: Supplementary file 3 — Additional file 3: Figure S3. DMSCs could be induced to differentiate as osteogenic cells or adipogenic cells. (A) osteogenic cells. Alizarin red stain. (B) adipogenic cells. Oil red stain. Bar = 100μm. (C) Growth curve of DMSCs. The initial cell number was 1×10^4^/well, and the exponential growth phase doubling speed was about 28h, stabilizing after 12 days. The results shown are means ± SEM. [file 13048_2022_993_MOESM3_ESM.tif]
